# Supplementary material for: The greenbeard gene tgrB1 regulates altruism and cheating in Dictyostelium discoideum
Source: Nat Commun. 2024 May 11;15:3984. doi: 10.1038/s41467-024-48380-4 (PMC11088635; doi:10.1038/s41467-024-48380-4)
Supplement: Supplementary file 1 — Supplementary Information [file 41467_2024_48380_MOESM1_ESM.pdf]

The greenbeard gene *tgrB1* regulates altruism and cheating in *Dictyostelium discoideum*

Mariko Katoh-Kurasawa, Peter Lehmann, and Gad Shaulsky

Supplementary Information

This document contains four supplemental figures and two supplemental tables.

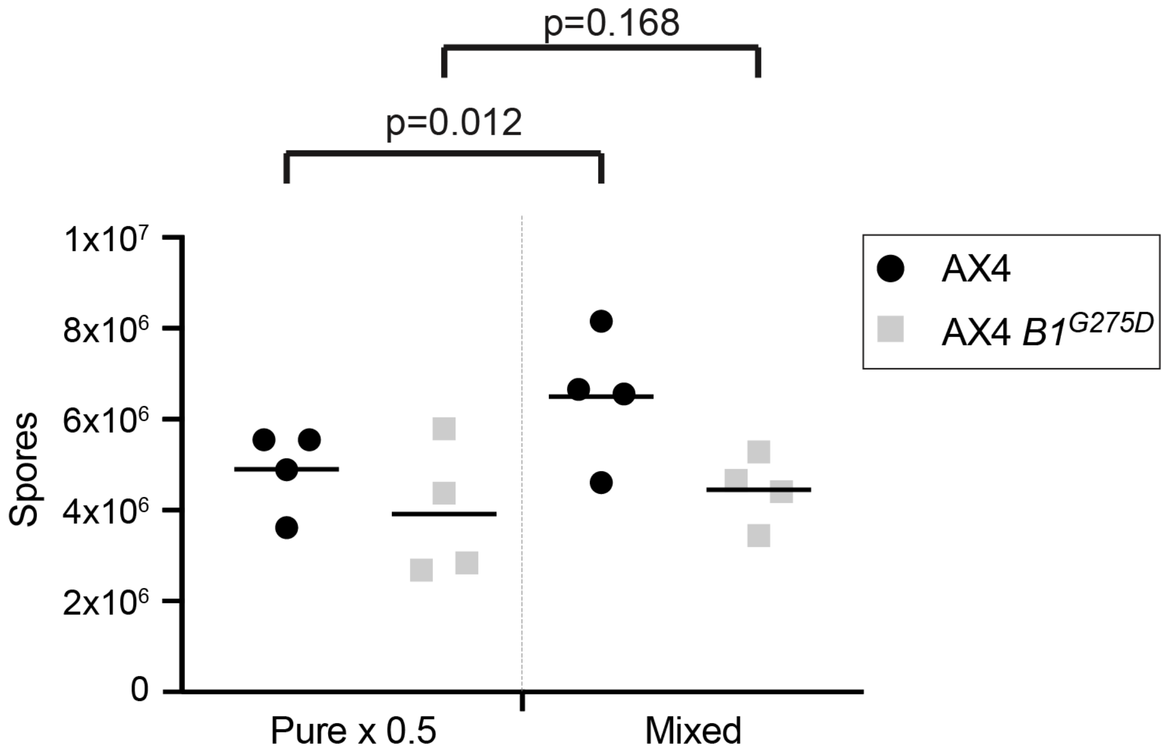

Supplementary Figure 1. Activated *tgrB1* confers altruistic behavior. We used two strains that express constitutive fluorescent markers, the wild-type AX4-GFP and the activated *tgrB1* mutant AX4 *tgrB1*^{G275D}-RFP. We grew the cells separately, developed 7x10^{6} cells either in pure populations or mixed at equal proportions as indicated, and counted spores. The spore counts are shown as four independent replicates (symbols) and their averages (horizontal lines). The pure population counts were multiplied by 0.5 to scale them with the mixed population. Brackets and p-values (T-test, one sided, n=4) compare the spore counts of each strain in the two conditions. Source data are provided as a Source Data file.

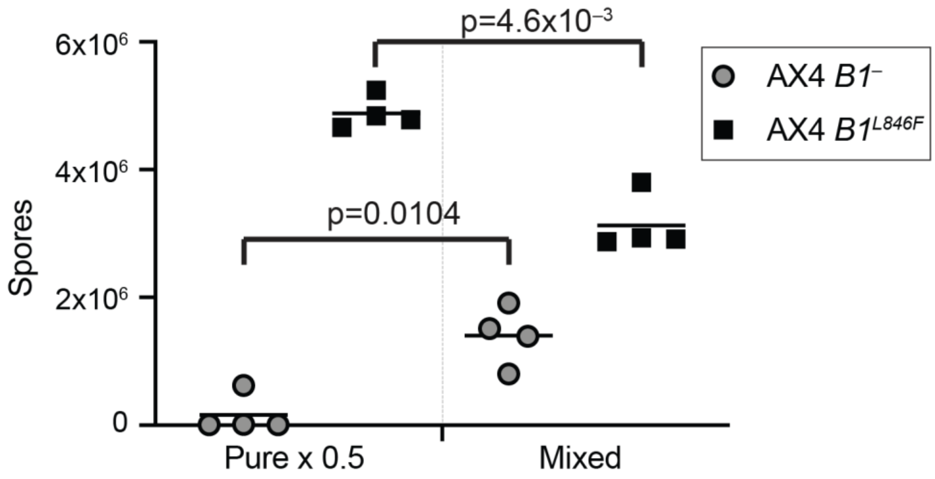

Supplementary Figure 2. Victim-cheater interactions between activated *tgrB1* and *tgrB1*⁻ cells. We used two strains that express constitutive fluorescent markers, the activated *tgrB1* mutant AX4 *tgrB1*^{L846F}-RFP and inactivated *tgrB1* mutants (*tgrB1*⁻-GFP). We grew the cells separately, developed 7x10^{6} cells either in pure populations or mixed at equal proportions as indicated, and counted spores. The spore counts are shown as four independent replicates (symbols) and their averages (horizontal lines). The pure population counts were multiplied by 0.5 to scale them with the mixed population. Brackets and p-values (T-test, one sided, n=4) compare the spore counts of each strain in the two conditions. Source data are provided as a Source Data file.

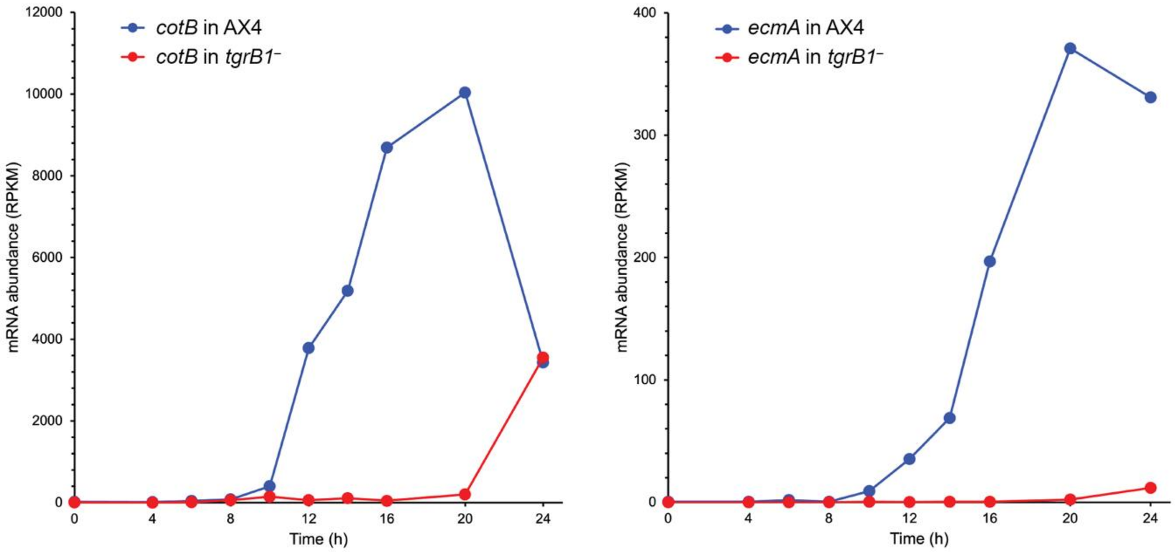

Supplementary Figure 3. Cell-type marker expression in AX4 and *tgrB1*⁻ cells. Gene expression data from our published RNA-sequencing database[38,39] describing the prespore marker *cotB* (left) and the prestalk marker *ecmA* (right) in the AX4 wild type (blue) and the *tgrB1*⁻ mutant (red). We plotted the mRNA abundance (RPKM – Reads Per Kilobase per Million, y-axis) as a function of developmental time (x-axis, hours). Expression of the cell-type markers is markedly reduced in the *tgrB1*⁻ mutant compared to the wild type. The images shown in Fig. 3 correspond to the 16-hour time point (finger stage) in this figure, in which the *tgrB1*⁻ mutant cells do not express detectable mRNA levels of the two markers. Source data are provided as a Source Data file.

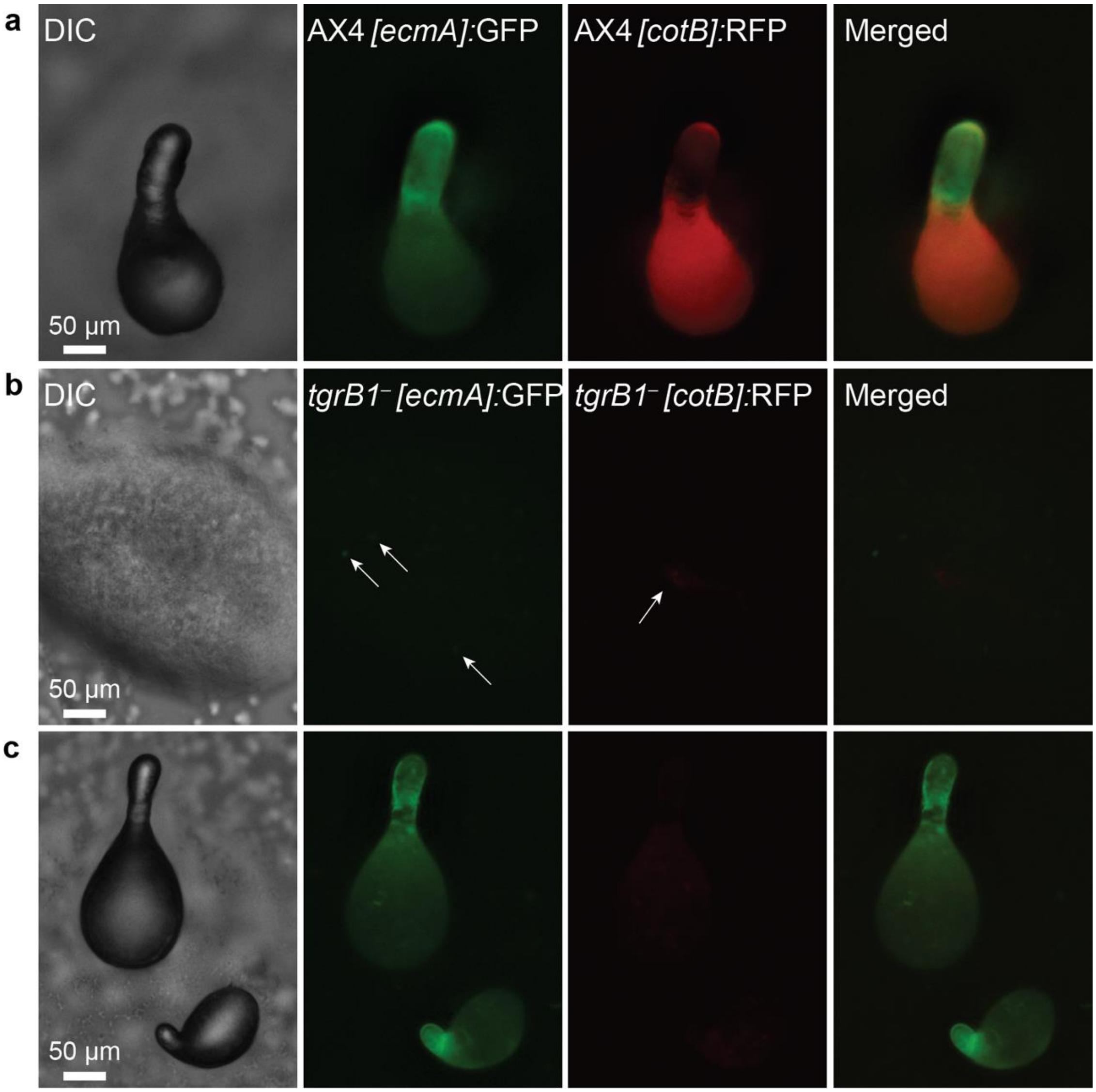

Supplementary Figure 4. Prestalk and prespore marker expression in pure populations. We used wild-type (AX4) and mutant (*tgrB1*$^{-}$) cells, both carrying the prestalk reporter *[ecmA]*:GFP and the prespore reporter *[cotB]*:RFP. We grew and developed the cells in pure populations and imaged at the finger stage. **a.** A typical early finger-stage of AX4 *[ecmA]*:GFP *[cotB]*:RFP. **b.** A typical loose aggregate of *tgrB1*$^{-}$ *[ecmA]*:GFP *[cotB]*:RFP. The white arrows indicate rare cells that express the respective markers. **c.** A rare finger-like structure of *tgrB1*$^{-}$ *[ecmA]*:GFP *[cotB]*:RFP at a late developmental time. We imaged the structures with DIC and with green and red fluorescence, and generated a merged image of the red and green channels, as indicated (the indications in c are the same as in b). Camera settings are included in Supplement File S1.

Supplementary Table 1. ***D. discoideum*** **Strains**

**Strain name (Dicty stock center ID)**	**Description**	**Parental strain**	**Antibiotic resistance and/or other selections**	**Reference**
AX4 (DBS0235552)	Laboratory wild type, axenic	NC4	N/A	Knecht et al., 1986
AX4-GFP	Constitutive expression of GFP under *act15* promoter	AX4	G418	Benabentos et al., 2009
AX4-RFP	Constitutive expression of mCherry under *act15* promoter	AX4	G418	This study
AX4-CFP	Constitutive expression of mCerulean under *act15* promoter	AX4	G418	This study
AX4-R/G	Expression of mCherry under the prespore promoter of *cotB* and mNeon Green under the prestalk promoter of *ecmA* (co-transformed)	AX4	G418	This study
AX4-R/Y	Expression of mCherry under the prespore promoter of *cotB* and YFP under the prestalk promoter of *ecmA* (single vector)	AX4	Hygromycin	This study
AX4-*tgrB1*^{L846F} RFP	Expression of the constitutively active allele *tgrB1*^{L846F} under the *tgrB1* promoter in AX4-RFP	AX4-RFP (AK1502)	Blasticidin S; G418	This study
AX4 *tgrB1*^{L846F} R/G	Expression of the constitutively active allele *tgrB1*^{L846F} under the *tgrB1* promoter in AX4-R/G	AX4-R/G	Blasticidin S; G418	This study
AX4-*tgrB1*^{G275D} RFP	Expression of the constitutively active allele *tgrB1*^{G275D} under the *tgrB1* promoter in AX4-RFP	AX4-RFP (AK1502)	Blasticidin S; G418	This study
AX4 *tgrB1*$^{-}$ GFP (DBS0307357)	Expression of GFP in AX4 in which the *tgrB1* gene was replaced with a	*tgrB1*$^{-}$ (DBS0304820)	Blasticidin S; G418	Benabentos et al., 2009

	BSR cassette (between 6-2,917 bp) by homologous recombination			
AX4 *tgrB1*⁻ GFP #2.1	Insertion of 28-bp following nucleotide 742 of *tgrB1*, generated by CRISPR/Cas9 mutagenesis	AX4-GFP	G418	This study
AX4 *tgrB1*⁻ GFP #2.4	Deletion of 1-bp at 745 bp of the *tgrB1* coding sequence, generated by CRISPR/Cas9 mutagenesis	AX4-GFP	G418	This study
AX4 *tgrB1*⁻ RFP	Expression of mCherry in AX4 in which the *tgrB1* gene was replaced with a Hygromycin resistance cassette (between 6-2,917 bp) by homologous recombination	*tgrB1*⁻ (DBS0349724)	Hygromycin; G418	This study
AX4 *tgrB1*⁻ CFP	Insertion of 20-bp following nucleotide 746 of the *tgrB1* coding sequence, generated by CRISPR/Cas9 mutagenesis	AX4-CFP	G418	This study
AX4 *tgrB1*⁻ R/G	Insertion of 4-bp following nucleotide 746 of the *tgrB1* coding sequence, generated by CRISPR/Cas9 mutagenesis	AX4-G/R	G418	This study
AX4 $B1^{\Delta}C1^{\Delta}$ GFP (DBS0349732)	Replacement of the *tgrB1-tgrC1* locus with *pyr5-6*	AX4 $B1^{\Delta}C1^{\Delta}$ (DBS0349731)	G418	Hirose et al., 2011
AX4 $B1^{AX4}C1^{AX4}$ RFP (DBS0349736)	Replacement of the resident *tgrB1-tgrC1* locus with the *tgrB1-tgrC1* locus from AX4	AX4 $B1^{AX4}C1^{AX4}$ (DBS0349734)	*pyr5-6*⁻; G418	Hirose et al., 2011
AX4 $B1^{QS31}C1^{QS31}$ RFP (DBS0349747)	Replacement of the resident *tgrB1-tgrC1* locus with the *tgrB1-tgrC1* locus from QS31	AX4 $B1^{QS31}C1^{QS31}$ (DBS0349738)	*pyr5-6*⁻; G418	Hirose et al., 2011

AX4 $B1^{\Delta}C1^{AX4}$ GFP	Expression of *tgrC1* from AX4 under its own promoter in the *tgrB1-tgrC1* double null strain, $B1^{\Delta}C1^{\Delta}$ GFP	AX4 $B1^{\Delta}C1^{\Delta}$ GFP (DBS0349732)	Blasticidin S; G418	This study
AX4 $B1^{\Delta}C1^{QS31}$ GFP	Expression of *tgrC1* from QS31 under *tgrC1*^{AX4} promoter in *tgrB1-tgrC1* double null strain, AX4 $B1^{\Delta}C1^{\Delta}$ GFP	AX4 $B1^{\Delta}C1^{\Delta}$ GFP (DBS0349732)	Blasticidin S; G418	This study

Supplementary Table 2: Oligonucleotides

**Strain**	**Oligonucleotide name**	**Sequence**
tgrB1^{-} (CRISPR)	tgrB1_N_F3<br>or<br>tgrB1_N_F5	5’ AAGTCTCAAT ATGTGGCTC 3’<br>or<br>5’ TTTCAATATT AGTAGTAGTG G 3’
	tgrB1_N_R3	5’ ATTGTATTTG ATTTATATTC ACC 3’
	Sequencing primer 1	5’ ATTGTATTTG ATTTATATTC ACC 3’
tgrB1^{L846F}	tgrB1_C_F4	5’ TATCTCTGTT GATGGTCAG 3’
	tgrB1_C_R4	5’ TAGAAGGTAC TTTTATCTGG 3’
	Sequencing primer 2	5’ TATAGTGAAA ACAAATCAAC AGG 3’
tgrB1^{G275D}	tgrB1_N_F3	5’ AAGTCTCAAT ATGTGGCTC 3’
	tgrB1_N_R3	5’ ATTGTATTTG ATTTATATTC ACC 3’
	Sequencing primer 1	5’ ATTGTATTTG ATTTATATTC ACC 3’
tgrB1 allotype	tgrB1(AX4)_spe_F2<br>or<br>tgrB1(QS31)_spe_F2	5’ GGATTCAATT ATGACCGTTG 3’<br>or<br>5’ CGTAATTGAA GGTAGACATG 3’
	tgrB1_ter_R2	5’ TATTGTAGAA TGAAAGTTGA GG 3’
tgrC1 allotype	tgrC1(AX4)_spe_F1	5’ TCAGGATATT CAATGAATCC 3’
	tgrC1(AX4)_spe_R1	5’ CAGTATTTGA TTTATCTCTA AC 3’
	tgrC1(QS31)_spe_F1	5’ GATTTCCAAT GCCTCGC 3’
	tgrC1(QS31)_spe_R1	5’ CAACCGGGCT TGATACT 3’
	tgrC1_common_F	5’ CTTTTGACTG TAATAATATT AC 3’
	tgrC1_common_R	5’ CAAGATTTGT ACCAATTAAT G 3’
	Sequencing primer 3	5’ CAAGATTTGT ACCAATTAAT G 3’
