# Supplementary material for: The greenbeard gene tgrB1 regulates altruism and cheating in Dictyostelium discoideum
Source: Nat Commun. 2024 May 11;15:3984. doi: 10.1038/s41467-024-48380-4 (PMC11088635; doi:10.1038/s41467-024-48380-4)
Supplement: Supplementary file 4 — Description of Additional Supplementary Files [file 41467_2024_48380_MOESM4_ESM.pdf]

Description of Additional Supplementary Files

File Name: Supplementary Data 1

Description: Camera Settings for all the micrographs shown in the main text and in the supplementary information, listed by figure names and subpanels.
